# Alloantibody Identification: The Importance of Temperature, Strength Reaction and Enzymes—A Practical Approach

**DOI:** 10.3390/hematolrep16040077

**Published:** 2024-12-17

**Authors:** Palma Manduzio

**Affiliations:** Diagnostic Department, Immunohematology and Transfusion Medicine, Policlinico Riuniti, Via Pinto 1, 71122 Foggia, Italy; ina.m77@alice.it; Tel.: +39-0881-732190

**Keywords:** red blood cell alloimmunization, antibody identification, transfusion safety

## Abstract

Red blood cell (RBC) alloimmunization and antibodies formation against non-self antigens on red cells may occur after blood transfusion, pregnancies or other exposures. The RBC alloimmunization rate varies from 2% to 6% according to recent studies. The antibody screen is performed to identify or confirm the presence of antibodies in patient’s serum or plasma, as a preoperative or pretransfusion test. The antibody identification process and major crossmatch are critical steps of risk management in transfusion medicine. The aim of this article is to describe a flow chart of the antibody identification. I report three educational examples of case studies associated with the negative direct antiglobulin test and clinically significant single and multiple alloantibodies using the gel method, Anti-M, Anti-c and Anti-E, Anti-Jka and Anti-s. Furthermore, I provide a critical analysis of the current literature on the topic. The flow chart of the antibody identification may simplify the process and possibly reduce errors in routine workflow.

## 1. Introduction

Blood group antigens are sugars or proteins attached to the red blood cell (RBC) membrane. Antibodies are immunoglobins produced by lymphocytes and plasma cells following antigen stimulation and are found in serum or in body fluids. They can be demonstrated serologically. Antibodies against red blood cells can be natural (e.g., Anti-A, Anti-B) or immune immunoglobulins (e.g., Anti-D or Anti-Fya). The former are mainly the IgM class with large molecular weight, capable of binding complement cascade and causing intravascular hemolysis; the latter are often IgG, capable of crossing the placenta and causing hemolytic disease of the fetus and newborn (HDFN) [1,2].

Some blood group antibodies are agglutinins, whereas others are hemolysins. Many factors influence antigen–antibody reaction, such as temperature, incubation time, pH, ionic strength of environment, zeta potential between red cells, concentrations of antigen and antibody and centrifugation [1,2].

Hemagglutination occurs when IgM react with their corresponding red cell antigens, and sensitization occurs when IgG react with their red cell antigens. The latter is not an observable reaction without potentiators such as Antihuman globulin (AHG), which contains IgG and complement fractions. Hemolysis is the result of antigen–antibody reaction that utilizes the complement proteins to mediate red cell membrane attack and lysis [1,2].

The indirect antiglobulin (Coombs) test is used to determine whether serum or plasma contains IgG non-ABO antibodies (antibody screen) and their identification. The sensitization of red cells is observed in vitro by incubating the red cells with the corresponding antibody from patient serum or plasma at 37 °C or room temperature (RT), with AHG and other potentiators (such as low-ionic-strength solution (LISS), enzymes) [1,2,3,4].

The direct antiglobulin test (DAT) is performed to detect IgG or complement proteins bound to patient red cells with AHG without incubation time and other potentiators [4].

Autocontrol (AC) tests the patient’s serum or plasma with their own red cells. It includes a potentiator (such as LISS, enzymes) and an incubation step at 37 °C or RT, similarly to the antibody screen and antibody identification [4].

Anemia is a global public health problem according to the World Health Organization (WHO). It is a condition in which the number of red cells or hemoglobin concentration is lower than normal. It is a treatable condition with different aetiologies [4]. RBC transfusion is a key component of therapy for medical and surgery patients and for chronic treatment of hematologic malignancies and hemoglobinopathies. After exposure to non-self-proteins or non-self-carbohydrates of RBCs, B cells are activated from the primary to secondary immune response. The former is T-cell-independent, with a slow and weak response and the latter is T-cell-dependent, with a strong and rapid response [5,6]

Antibody formation against non-self antigens on red blood cells may occur after transfusions, pregnancies or other exposures. Multiple triggers for red blood cell alloimmunization include donor and recipient factors (e.g., ethnicity, clinical conditions) and red blood cell factors (e.g., antigen variants, immunogenicity) [7,8,9].

The RBC alloimmunization rate varies from 2% to 6% according to recent studies [10]. The Recipient Epidemiology and Donor Evaluation Study-III (REDS-III), which included 319.177 recipients, recognized that older age, female sex, Rh (D)-negative status, hemoglobinopathies and autoimmune disorders were risk factors for alloimmunization [10].

More than 100 million RBC units are collected worldwide each year [11]. RBCs are biological products with variability and different transfusion effectiveness [12,13]. Furthermore, studies of transfusion exposure and patient outcome are limited by confounding factors and possibly biased methods [14]. Nowadays, the safety of RBC products depends on immunological compatibility, substantially [15].

Therefore, considering the complex interaction among RBCs, donors and recipients, the genetics of blood group antigens and clinical conditions, further studies are necessary to better analyze the risk factors for alloimmunization and improve the ability to supply compatible red blood units [9].

## 2. Materials and Methods

A type and screen sample, the ethylenediaminetetraacetic acid (EDTA) anticoagulant, is submitted to the blood bank with a request for units of red blood cells.

The antibody screen is performed by mixing patient’s plasma or serum with three reagent red cells of blood type O, with phenotypes for most common antigen specificities. RBCs and plasma or serum are incubated, centrifuged and interpreted based on the degree of agglutination (from 0 to 4+, no reaction versus strong reaction) and hemolysis.

A negative antibody screen suggests an absence of antibodies or that they are undetectable. In case of a positive antibody screen, a more complete antibody panel of 11 reagent red cells of blood type O is provided to detect non-ABO antibodies. Assigning presumptive antibody specificity is useful to select appropriate red blood units and determine the compatibility of donor red cells with patient’s serum or plasma (major crossmatch).

A positive autocontrol and a negative DAT may indicate a false positive result. A negative autocontrol may exclude autoantibodies, drug interaction, and transfused cells sensitized with antibodies [4,15].

The antibody identification is a multistep process [4,15]. Firstly, the temperature evaluation of the antigen–antibody reaction (for example, reaction at 37 °C suggests clinically significant IgG antibody; reaction at room temperature may suggest irrelevant IgM antibody) and the strength reaction (for example, different scores of hemagglutination may be due to multiple antibodies or antibody with dosage) are important; secondly, cross out the antibody.

More specifically, the laboratory protocol of exclusion is based on observation of antigens present on RBC reagents of the panel with which patient serum or plasma did not react. For antibodies which demonstrate dosage (Rh excluding D, Kidd, Duffy, MNS blood groups), caution should be exercised when ruling out panel cells that have homozygous antigen expression.

Furthermore, crossing out the antibody for antigens D, C, E, c, k, M, N, S, s, Fya, Fyb, Jka, and Jkb is based on a minimum of two non-reactive cells with homozygous antigen expression, while the exclusion of anti-K is based on one non-reactive cell with homozygous antigen expression or two non-reactive cells with heterozygous antigen expression [4,15].

Finally, it is important to detect three antigen-positive cells to be reactive and three antigen negative cells to be non-reactive to assign presumptive antibody specificity (‘Rule of Three’), match the pattern of reaction and confirm correlation with the patient’s phenotype [4,15].

**Figure 1:**
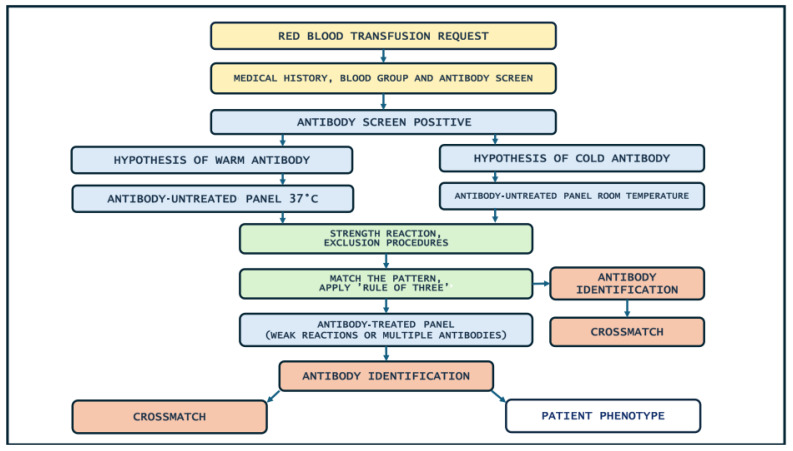
Flow chart of antibody identification.

**Figure 2:**
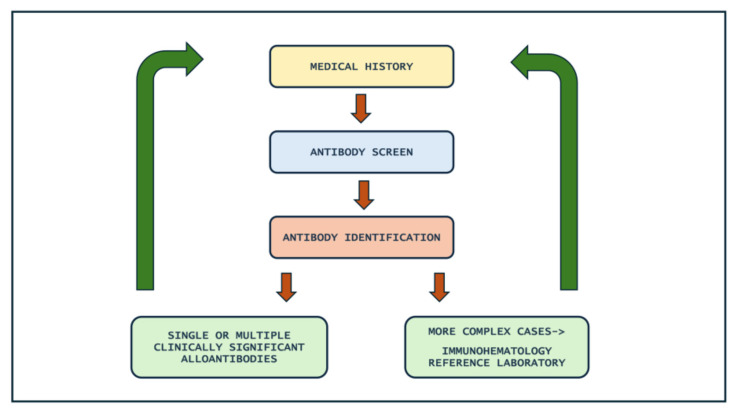
Steps of antibody identification.

Figure 1 illustrates the flow chart of the antibody identification to follow in routine workflow in case of a positive antibody screen, negative autocontrol and negative direct antiglobulin test. Figure 2 describes the steps of the antibody identification process.

## 3. Results

### 3.1. Example 1 and the Importance of Temperature

Table 1 represents the ABO/Rh (D) type. The forward type of the patient’s EDTA sample using the gel method (microtube filled with gel particles and reagent Anti-A, Anti-B and Anti-A, B) indicates that the patient’s group is A; in fact, a 0.8%-1% red cells suspension in saline is reactive with Anti-A due to the A antigen on the red cell membrane. The reverse type of the sample (using A1 cells, B cells and O cells) confirms that anti-B isoantibodies are detected in the plasma. Forward results are consistent with reverse results.

In a similar manner, testing with reagent anti-D recognizes that the Rh(D) is present on the patient’s red cells. In conclusion, the patient’s group is A Rh-positive.

Table 2 summarizes the antibody screen using the gel method. Alternatively, the reaction may be performed using tube test or solid phase technology [16].

Table 3 summarizes the antibody identification panel using the gel test with untreated RBC reagents. The *Anti-M antibody* can be identified, and other clinically significant alloantibodies can be ruled out. This antibody is active at 37 °C and expresses a dosage effect [15,17].

In fact, the reaction is stronger with homozygous M antigen cells than with eterozygous M antigen cells (M + N− reagent red cells versus M + N+ reagent red cells). Most antibodies to the M antigen are cold reacting and they are not considered clinically significant [18]. Dithiothreitol (DTT) is a reducing agent used to differentiate the IgM and IgG reactivity of the antibody. This case suggests a warm reacting alloantibody anti-M [19,20,21,22].

Compatible RBC units should be provided through the indirect antiglobulin test (IAT) [15].

In addition, the serologic phenotype may confirm identification of the antibody. In case of pregnancy, IgG alloanti-M should be confirmed using a different method such as the tube test or solid phase technology and anti-M titer should be monitored for prevention of hemolytic disease of the fetus and newborn [23].

### 3.2. Example 2 and the Importance of Strength Reaction

Table 4 presents the ABO/Rh (D) type. The forward type of patient’s sample using the gel method is A, Rh-positive. Forward results are consistent with reverse results. Table 5 presents the antibody screen. Table 6 and Table 7 summarize the antibody identification panel using the gel test with two different untreated RBCs.

Alloantibodies against the Rh (D) are confirmed in the gel panel. Interestingly, a variety of strength reactions may suggest the presence of multiple antibodies [15].

*Anti-c and Anti-E* can be identified, and all other clinically significant alloantibodies can be ruled out (Table 6 and Table 7) [15].

Rh blood group system antibodies are commonly observed after transfusion or pregnancy. They show specific characteristics; most are IgG, and bind at 37 °C on the indirect antiglobulin test (IAT). Some Rh antibodies may be found in individuals who have never been pregnant (Anti-Cw) or undergone transfusion (Anti-E). After alloimmunization, antibodies may persist for several years. Furthermore, the Rh(c) antigenic is more immunogenic than the Rh(E) antigen and may cause severe HDFN [24,25,26,27].

A low titer of Anti- E and Anti-c is recognized using the gel method. Potentiators such as enzymes or a more sensitive method of antibody identification may be used in case of weak reactions [15].

Ultimately, c-negative and E-negative blood units should be provided and crossmatched [15].

### 3.3. Example 3 and the Importance of Enzymes

Table 8 presents the ABO/Rh (D) type. The forward type of the patient’s sample using the gel method is B, Rh-positive. Forward results are consistent with reverse results.

Table 9 presents the antibody screen. Table 10 summarizes the antibody identification panel using the gel test with untreated RBCs and enzyme (ficin)-treated red blood cells.

Multiple antibodies need a selected panel of red blood cells and additional techniques such as enzymes. More specifically, antibody reactions are enhanced using enzymes in the Rh, P1, I, Kidd and Lewis blood groups. Antigens destroyed by enzymes are M, N, and Duffy. The Kell blood group is unaffected by the enzyme (ficin) reagent. S and s antigens present a variable effect on enzymes [15,17]. In addition, matching the pattern is more difficult when more than one antibody specificity exists.

*Alloantibodies against the Fya and s* are confirmed in the gel panel and all other clinically significant alloantibodies are ruled out (Table 10) [15].

Duffy antibodies are observed after acute and delayed transfusion reactions and are an uncommon cause of HDFN. Anti-Fya is IgG; it does not usually bind the complement proteins. The antibody is classically non-reactive with ficin-treated RBC reagents because the enzyme degrades the antigen. Anti-s is a clinically significant IgG antibody that can induce hemolysis and HDFN. Anti-s shows a variable effect after ficin-treated red blood cells [4,18].

Fya-negative and s-negative red blood units should be provided and crossmatched for transfusion requests [15].

## 4. Discussion

One of the most critical processes in transfusion medicine is the identification of single or multiple clinically significant alloantibodies by a laboratory protocol.

Figure 1 and Figure 2 summarize the identification antibody process and a multistep approach. It may simplify and possibly reduce trivial errors in workflow.

Proper management of patients includes major compatibility of RBC products, collection and comparison of immunohematology tests, explanatory notes and clear communication with clinicians.

The antibody screen is performed to identify or confirm the presence of antibodies in the patient’s serum or plasma, as preoperative and pretransfusion tests. Antibody identification and major crossmatch are subsequently provided using the indirect antiglobulin test [15].

The antibody screen is summarized in Table 2, Table 5, and Table 9. The antibody panel is summarized in Table 3, Table 6, Table 7 and Table 10. The three examples describe significant single and multiple alloantibodies in clinical practice. Antibodies to low-prevalence antigens are not reported in the manuscript. In fact, more complex cases may benefit from different reagent panel cells and methods, different potentiators and specific analysis of a local immunohematology reference laboratory (IRL).

Anti-M, documented in the first example, is a possible warm reacting antibody. For decision-making during pregnancy, it is of paramount importance to obtain a complete transfusion history and monitor the antibody titer accurately.

Alloantibodies to the Rh antigen are observed in the second example. Potentiators such as enzymes or a more sensitive method of antibody identification are necessary to increase weak reactions. In addition, matching the pattern is more difficult when more than one antibody specificity exists.

Anti-s, recognized in the last example, is IgG and reactive at 37 °C. Similarly, Anti- Fya is immunogenic and known to cause a hemolytic reaction and HDFN. The Duffy system includes antigens such as Fya and Fyb. Caucasian individuals express some combination of Fya and/or Fyb, while blacks may express neither antigen. Eventually, it is important to collect information regarding ethnicity, because it can expedite the search for red blood units and a supply of compatible products [4].

## 5. Conclusions

The transfusion staff recommend red blood units for transfusion requests based on medical history and immunohematology tests (blood group, antibody screen and identification).

A multistep flow chart of the antibody identification may be useful to improve transfusion safety, reduce risk for transfusion reactions and hemolysis and improve transfusion effectiveness. In addition, it may simplify the process and possibly reduce trivial errors in workflow.

In summary, of paramount importance is the evaluation of temperature and score of hemagglutination, such as a further treated panel of red blood cells in some complex cases. In fact, more complex cases need studies from an IRL.

Ultimately, communication between the transfusion specialist and clinician is necessary to expedite the search for red blood units and proper management of the patient [28,29,30,31].

## Figures and Tables

**Table 1:** AB0/Rh (D) gel method.

**RBCs Forward Type**			
Anti-A	Anti-B	Anti-A, B	Anti-D
4+	0	4+	4+
**Plasma Reverse Type**			
A1 cells	B cells		0 cells
0	4+		0

Reaction scale = 0 (no reaction) to 4+ (strong reaction); RBCs: red blood cells.

**Table 2:** Antibody screen.

Cell	D	C	E	c	e	f	K	k	Fya	Fyb	Jka	Jkb	Lea	Leb	P1	M	N	S	s	Gel
1	+	+	0	0	+	0	+	+	0	+	+	0	0	+	+s	0	+	0	+	0
2	+	0	+	+	0	0	0	+	+	0	+	+	0	+	+	+	0	+	0	2+
3	0	0	0	+	+	+	0	+	+	+	0	+	+	0	+	+	+	0		0

Reaction scale = 0 (no reaction) to 4+ (strong reaction), +s: strong reaction; Gel/IAT: antiglobulin test.

**Table 3:** Antibody identification panel with untreated RBCs.

Cell	D	C	E	c	e	f	K	k	Fya	Fyb	Jka	Jkb	Lea	Leb	P1	M	N	S	s	Gel
1	+	+	0	0	+	0	0	+	+	+	0	+	0	+	+	+	0	+	+	2+
2	+	+	0	0	+	0	0	+	0	+	+	+	+	0	+	+	+	+	0	0
3	+	0	+	+	0	0	0	+	+	+	+	0	0	0	+	0	+	0	+	0
4	+	0	0	+	+	+	0	+	+	0	0	+	0	+	0	+	+	0	+	0
5	0	+	0	+	+	+	0	+	+	+	0	+	0	+	+	+	+	0	+	0
6	0	0	+	+	+	+	0	+	0	+	0	+	0	+	+	+	+	0	+	0
7	0	0	0	+	+	+	0	+	0	+	+		0	+	0	+	0	0	+	2+
8	0	0	0	+	+	+	0	+	+	0	+	+	+	0	+	+	0	+	0	2+
9	0	0	0	+	+	+	0	+	+	0	+	0	0	+	+	0	+	+	+	0
10	0	0	0	+	+	+	0	+	+	0	+	+	0	+	+	0	+	0	+	0
11	+	+	0	0	+	0	+	+	0	+	0	0	0	+	+	+	0	+	0	1+
Pat. Cells	+	+	0	+	+											0	+	0	+	AC: neg

Pat: patient; reaction scale = 0 (no reaction) to 4+ (strong reaction); AC: autocontrol; neg: negative; Gel/IAT: antiglobulin test; RBCs: red blood cells.

**Table 4:** AB0/Rh (D) gel method.

**RBCs Forward Type**			
Anti-A	Anti-B	Anti-A,B	Anti-D
4+	0	4+	4+
**Plasma reverse type**			
A1 cells	B cells		0 cells
0	4+		0

Reaction scale = 0 (no reaction) to 4+ (strong reaction); RBCs: red blood cells.

**Table 5:** Antibody screen.

Cell	D	C	E	c	e	f	K	k	Fya	Fyb	Jka	Jkb	Lea	Leb	P1	M	N	S	s	Gel
1	+	+	0	0	+	0	0	+	0	0	+	+	+	0	+s	+	0	+	0	0
2	+	0	+	+	0	0	+	+	+	+	0	+	0	0	+	+	0	+	+	0.5+/1
3	0	0	0	+	+	+	0	+	+	+	+	0	+	+	+s	0	+	0	+	1+

Reaction scale = 0 (no reaction) to 4+ (strong reaction), +s: strong reaction; Gel/IAT: antiglobulin test.

**Table 6:** Antibody identification panel with untreated RBCs.

Cell	D	C	E	c	e	f	K	k	Fya	Fyb	Jka	Jkb	Lea	Leb	P1	M	N	S	s	Gel
1	+	+	0	0	+	0	0	+	+	+	+	+	0	+	+	+	+	+	+	0
2	+	+	0	0	+	0	0	+	0	+	0	+	0	+	+	+	0	+	0	0
3	+	0	+	+	0	0	0	+	0	+	0	+	0	+	+	+	+	0	+	1+
4	+	0	0	+	+	+	0	+	0	+	+	0	0	+	0	+	0	+	0	2+
5	0	+	0	+	+	+	0	+	+	0	+	+	0	+	+	+	0	+	0	0
6	0	0	+	+	+	+	0	+	+	+	+	+	0	+	+	0	+	0	+	2+
7	0	0	0	+	+	+	+	+	0	+	+	0	0	+	+	0	+	0	+	1+
8	0	0	0	+	+	+	0	+	+	0	0	+	+	0	0	+	+	+	+	2+
9	0	0	0	+	+	+	0	+	+	0	+	0	0	0	+	+	0	+	0	1+
10	0	0	0	+	+	+	+	+	+	+	0	+	0	+	+	0	+	+	+	1+
11	+	0	0	0	+	0	+	+	0	+	+	+	+	0	+	+	+	0	+	0
Pat. Cells	+	+	0	0	+		0	+												AC: neg

Pat: patient; reaction scale = 0 (no reaction) to 4+ (strong reaction); Gel/IAT: antiglobulin test; AC: autocontrol; neg: negative; RBCs: red blood cells.

**Table 7:** Antibody identification panel with untreated RBCs.

Cell	D	C	E	c	e	f	K	k	Fya	Fyb	Jka	Jkb	Lea	Leb	P1	M	N	S	s	Gel
12	0	0	0	+	+	+	+	+	+	0	+	0	0	0	+	0	+	0	+	1+
13	0	0	0	+	+	+	0	+	+	+	0	+	0	+	+s	+	0	+	0	1+
14	0	0	0	+	+	+	0	+	0	+	+	0	0	+	0	+	+	0	+	1+
15	0	0	+	+	0	0	0	+	+	+	+	0	0	0	+	+	0	+	0	1+
16	+	0	+	+	0	0	0	+	0	+	0	+	0	0	0	0	+	0	+	1+
17	+	0	+	+	0	0	0	+	+	0	+	+	0	0	0	+	+	0	+	1+
18	+	+	0	0	+	+	0	+	0	+	0	+	0	+	+	+	+	0	+	0
19	+	+	0	0	+	+	0	+	+	0	+	0	+	0	+s	0	+	0	+	0
20	+	+	+	0	+	+	0	+	+	+	0	+	0	0	0	0	+	+	0	0
21	+	0	+	+	+	+	0	+	+	0	0	+	0	0	+s	0	+	0	+	2+
22	0	+	0	+	+	+	+	0	+	+	+	0	+	0	+	+	+	0	+	0.5+ /1+
Pat. Cells	+	+	0	0	+		0	+												AC: neg

Pat: patient; reaction scale = 0 (no reaction) to 4+ (strong reaction); Gel/IAT: antiglobulin test; AC: autocontrol; neg: negative; RBCs: red blood cells.

**Table 8:** AB0/Rh (D) gel method.

**RBCs Forward Type**			
Anti-A	Anti-B	Anti-A,B	Anti-D
0	4+	4+	4+
**Plasma reverse type**			
A1 cells	B cells		0 cells
4+	0		0

Reaction scale = 0 (no reaction) to 4+ (strong reaction); RBCs: red blood cells.

**Table 9:** Antibody screen.

Cell	D	C	E	c	e	f	K	k	Fya	Fyb	Jka	Jkb	Lea	Leb	P1	M	N	S	s	Gel
1	+	+	0	0	+	0	0	+	+	+	+	+	0	+	0	+	0	+	+	0
2	+	0	+	+	0	0	0	+	+	0	+	0	0	+	+	0	+	0	0	3+
3	0	0	0	+	+	+	+	+	0	+	0	+	+	0	+	+	0	+	+	1+

Reaction scale = 0 (no reaction) to 4+ (strong reaction); Gel/IAT: antiglobulin test.

**Table 10:** Antibody identification panel with untreated and treated RBCs.

Cell	D	C	E	c	e	f	K	k	Fya	Fyb	Jka	Jkb	Lea	Leb	P1	M	N	S	s	Gel	Fic
1	+	+	0	0	+	0	0	+	+	0	+	0	0	+	+	+	+	0	+	1+	1+
2	+	+	0	0	+	0	0	+	0	+	+	+	0	+	0	0	+	+	0	0.5+/1	0.5+/1
3	+	0	+	+	0	0	+	+	0	+	0	+	0	+	+	+	+	0	+	0.5+/1	1+
4	+	0	0	+	+	+	0	+	0	+	+	0	0	0	0	+	+	0	+	0	0.5+/1
5	0	+	0	+	+	+	0	+	0	+	+	+	0	+	0	+	+	0	+	1+	0
6	0	0	0	+	+	+	0	+	+	+	+	+	+	0	+	+	0	+	+	1+	1+
7	0	0	0	+	+	+	+	+	0	0	+	0	0	+	+	0	+	0	+	0	1+
8	0	0	0	+	+	+	0	+	+	0	0	+	0	+	+	+	0	+	0	0	0
9	0	0	0	+	+	+	0	+	+	0	0	+	+	0	+	+	0	0	+	0	0
10	0	0	+	+	+	0	0	0	0	+	0	+	0	+	+	0	+	0	+	0	0
11	+	+	0	0	0	0	0	0	0	+	+	0	+	0	+	+	0	+	+	0.5+/1+	0.5+/1
Pat. Cells																				AC: neg	AC: neg

Pat: patient Reaction scale = 0 (no reaction) to 4+ (strong reaction); Gel/IAT: antiglobulin test; AC: autocontrol; neg: negative, Fic: ficina; RBCs: red blood cells.

## Data Availability

No data reporting.
